# Comparative analysis of cardiac mechano-energetics in isolated hearts supported by pulsatile or rotary blood pumps

**DOI:** 10.1038/s41598-019-56344-8

**Published:** 2019-12-27

**Authors:** Marcus Granegger, Young Choi, Benedikt Locher, Philipp Aigner, Emanuel J. Hubmann, Frithjof Lemme, Nikola Cesarovic, Michael Hübler, Martin Schweiger

**Affiliations:** 1 Pediatric Cardiovascular Surgery, Department of Surgery, Pediatric Heart Center, University Children’s Hospital Zurich, Zurich, Switzerland; 2 Children’s Research Center, University Children’s Hospital Zurich, Zurich, Switzerland; 3 Biofluid Mechanics Laboratory, Institute for Imaging Science and Computational Modelling in Cardiovascular Medicine, Charité-Universitätsmedizin Berlin, Berlin, Germany; 4 Center for Medical Physics and Biomedical Engineering, Medical University of Vienna, Vienna, Austria; 5 Ludwig Boltzmann Institute for Cardiovascular Research, Vienna, Austria; 6 Division of Surgical Research, Department of Surgery, University Hospital Zurich, University of Zurich, Zurich, Switzerland

**Keywords:** Circulation, Cardiac device therapy, Preclinical research

## Abstract

The previously more frequently implanted pulsatile blood pumps (PBPs) showed higher recovery rates than the currently preferred rotary blood pumps (RBPs), with unclear causality. The aim of this study was to comparatively assess the capability of PBPs and RPBs to unload the left ventricle and maintain cardiac energetics as a possible implication for recovery. An RBP and a heartbeat synchronized PBP were alternately connected to isolated porcine hearts. Rotational speed of RBPs was set to different support levels. For PBP support, the start of ejection was phased to different points during the cardiac cycle, prescribed as percentage delays from 0% to 90%. Cardiac efficiency, quantified by the ratio of external work over myocardial oxygen consumption, was determined. For RBP support, higher degrees of RBP support correlated with lower left atrial pressures (LAP) and lower cardiac efficiency (r = 0.91 ± 0.12). In contrast, depending on the phase delay of a PBP, LAP and cardiac efficiency exhibited a sinusoidal relationship with the LAP minimum at 90% and efficiency maximum at 60%. Phasing of a PBP offers the possibility to maintain a high cardiac efficiency and simultaneously unload the ventricle. These results warrant future studies investigating whether optimized cardiac energetics promotes functional recovery with LVAD therapy.

## Introduction

Mechanical circulatory support (MCS) has progressed from large extracorporeal pulsatile flow devices to smaller continuous flow rotary blood pumps (RBPs). The formerly more frequently used pulsatile blood pumps (PBPs) were gradually replaced with newly developed RBPs due to improved survival rates, smaller device sizes, and higher reliability^1^. In the last 10 years, the global number of implanted RBPs substantially exceeded the number of implanted PBPs, currently accounting for 97% of total implants^2,3^. Although the survival rates of MCS patients improved significantly during this period, the cardiac recovery rates remained sparse^4^. Krabatsch *et al*. observed an almost threefold higher chance of cardiac recovery in patients treated with PBPs compared to RBPs^5^. What causes these differences in cardiac recovery rates between RBPs and PBPs remains unclear.

Functional cardiac recovery for end-stage heart failure during left ventricular assist device (LVAD) support with subsequent device explantation constitutes a curative treatment option for the dysfunctional heart^6–8^. Therefore, the process of reversing the pathological morphology of failing hearts and how functional remodeling is induced, is of great interest. The adverse remodeling of the failing heart is primarily triggered by left ventricular pressure- or volume-overload^9,10^. This leads to several unfavorable intra- and extracellular changes in the myocardium such as cardiac hypertrophy, disturbed calcium homeostasis, and fibrosis^11^.

Recent studies suggest that ventricular unloading with MCS is the main stimulus that stops myocardial remodeling and reverses the negative changes to some extent^10,12–14^. Hence, the pursuit to delineate the optimal conditions for reverse remodeling and cardiac recovery with MCS has become an ongoing research topic and major focus in the scientific community^8,9^. Presently, it is debated whether prolonged MCS support with RBPs may not only lead to beneficial reverse remodeling but in parallel induce unfavorable myocardial atrophy^15,16^. The theory behind possible atrophy being that decreased myocardial energy demand due to excessive unloading causes myocardial hibernation and suppression of cellular repair^17^. This decline toward cardiac atrophy could possibly be prevented by providing a balanced degree of ventricular unloading that adequately unloads the LV without inducing the negative cascade toward atrophy.

A healthy cardiovascular system adapts the loading conditions and the ventricular properties to operate at a state of maximum cardiac efficiency, defined as the ratio between external ventricular work (EW) and myocardial oxygen consumption^18^. In the case of heart failure, the loading conditions with a highly elevated ventricular preload lead to a ventricle that no longer operates at the state of highest cardiac efficiency in order to provide sufficient cardiac output (CO) and systemic pressure^19^. These effects may be amplified by the fact that not only the boundary conditions are affected by chronic heart failure but also the ventricles’ peak efficiency is shifted to even lower preloads and afterloads^20^. The adversely altered loading situations cause a diminished ratio of useful mechanical work and consumed energy in the failing heart, which indicates that cardiac energetics are shifted to less efficient conditions.

To promote cardiac recovery, it may be beneficial to reestablish the operating condition of a healthy ventricle. This may be achieved by reducing the volume overload while simultaneously maintaining a high cardiac efficiency^21^. Whereas it has been previously shown how cardiac mechanics and hemodynamics are affected by RBPs and PBPs^22,23^, cardiac efficiency has not yet been investigated in a realistic setting. Therefore, the aim of this study was to comparatively assess cardiac hemodynamics, mechanics, and energetics between left ventricles supported by RBPs and PBPs in an *ex-vivo* isolated beating heart. We hypothesized that phasing of a PBP offers an additional degree of freedom to alter cardiac efficiency.

## Results

With the RBP implanted, in six isolated porcine hearts at least three speed settings were recorded with stable cardiac output, aortic pressure (AoP), and heart rate (HR, acceptance criteria ±10%) throughout the speed changes (Table 1). With the PBP implanted, in three experiments at least one complete cycle from 0% to 90% phase delay was successfully recorded with stable cardiac output, aortic pressure, and heart rate throughout each phase sweep (Table 1).

**Table 1:** Median and range of baseline hemodynamic parameters of the experiments with RBP and PBP support, n indicates the number of experiments.

	Heart Rate (bpm)	Cardiac Output (L/min)	Aortic Pressure (mmHg)
RBP support (n = 6)	Median 79 Range 50–103	Median 4.4 Range 3.3–5.0	Median 74 Range 58–81
PBP support (n = 3)	Median 88 Range 73–94	Median 4.1 Range 3.4–4.7	Median 67 Range 51–71

RBP – rotary blood pump; PBP – pulsatile blood pump.

### Hemodynamics with the RBP systems

**Figure 1:**
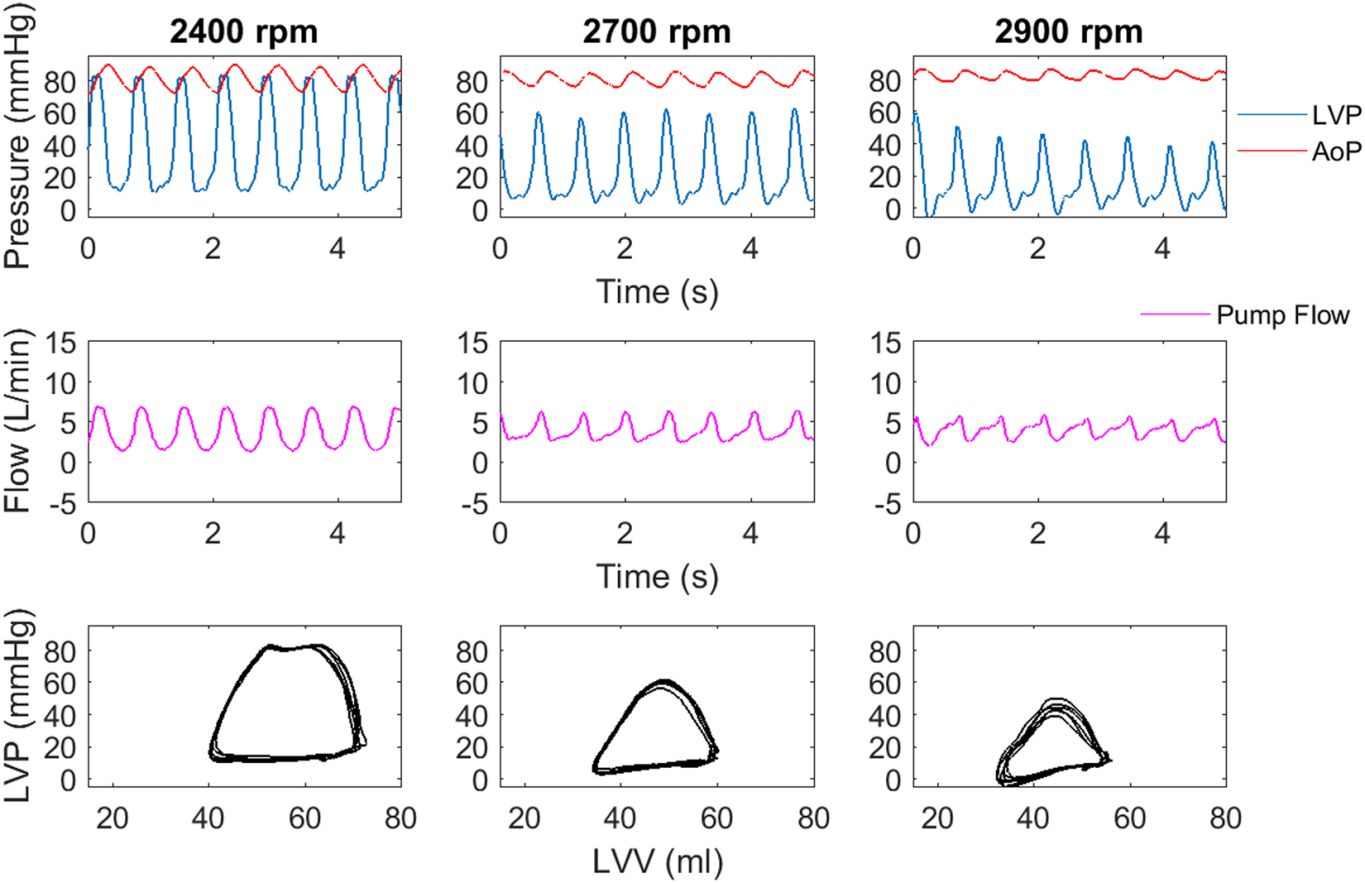
Typical time courses of pressure and flows at three different pumps speeds (upper and mid panel) at a cardiac output of 3.4 L/min. In the lower panel the respective PV loops are depicted.

Increasing pump speed of an RBP at constant cardiac output and arterial pressure lowered the ventricular preload, while simultaneously shifting the pressure – volume (PV) loop to lower volumes and decreasing EW (Fig. 1). Mean pump flow increased until the ventricle was fully supported (instantaneous left ventricular pressure (LVP) lower than the aortic pressure) and remained constant afterwards despite increasing pump speeds.

**Figure 2:**
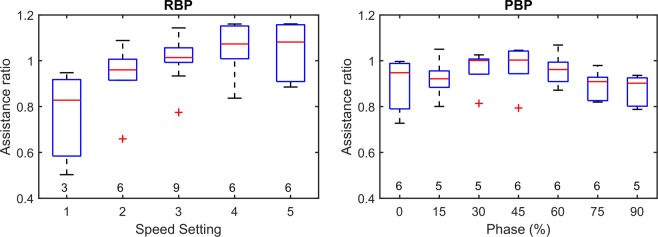
Box plots of assistance ratios recorded during RBP and PBP support. The sample size of each measurement is noted below each of the boxes.

The assistance ratio, defined as the ratio of mean pump flow in each support condition over the constant cardiac output, increased with increasing pump speeds (Fig. 2) indicating the transition of partial to full support at higher speed settings. For the purpose of statistical analysis, the speed setting was categorized into five ranges: (1) <2200 rpm, (2) 2200–2400 rpm, (3) 2400–2600 rpm, (4) 2600–2800 rpm, (5) >2800 rpm.

**Figure 3:**
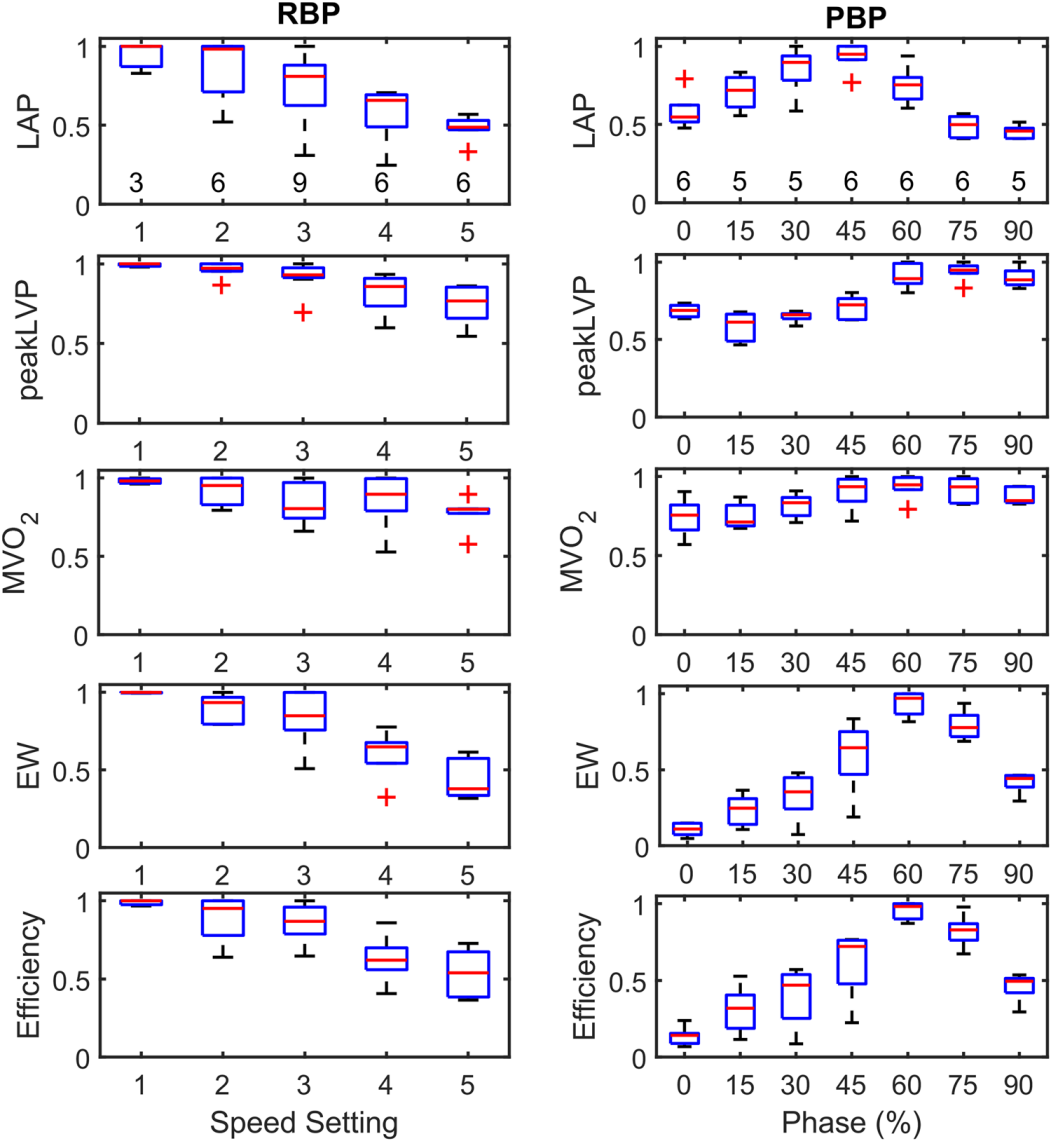
Box plots of hemodynamic parameters normalized by the maximum value of each experiment for the RBP and PBP. The number of included measurements in each of the box is presented in the upper panels.

Figure 3 (left) summarizes the relevant hemodynamic parameters of the six RBP experiments normalized by the maximum value for each experiment to compensate for inter-experimental variability. It can be observed that the median left atrial pressure (LAP), EW, and cardiac efficiency decreased by approx. 50% when comparing the lowest and highest speed setting; Median oxygen consumption decreased by approx. 20%. Peak LVP decreased once the aortic valve is closed during the entire heart cycle (full support at speed settings 4 and 5).

**Figure 4:**
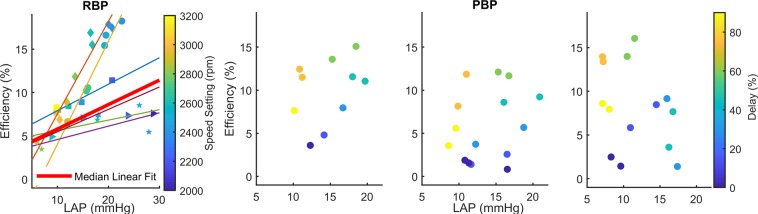
Relationship between LAP, pump speed, and cardiac efficiency for 6 experiments (visualized by different symbols) with an RBP. The linear regression relationship for each of the 6 experiments as well as the median linear fit is indicated (left); Relationship between LAP, cardiac efficiency, and phase delay for 3 experiments with a PBP (right). Starting in the left lower corner of each panel, the relationships develop in a counter-clock wise manner with increasing phase delay.

In each of the six experiments, cardiac efficiency and LAP correlated strongly (r = 0.91 ± 0.12). Linear regression analysis revealed a median slope [IQR] of 0.28 [0.91] %/mmHg and a median intercept [IQR] of 2.98 [7.52] %. Figure 4 (left) depicts the effect of increasing pump speed on LAP and cardiac efficiency for each of the 6 experiments.

### Hemodynamics with the PBP system

**Figure 5:**
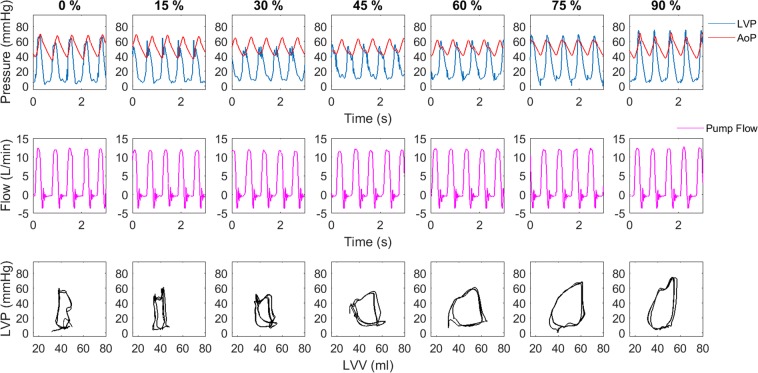
Typical time courses of pressure and flow at different phase delays (upper and mid panel) at a cardiac output of 3.4 L/min. In the lower panel the respective PV loops are depicted.

At a constant cardiac output, the lowest preload occurred during a phase delay of 75 to 90% (Fig. 5) Systolic LVP was highest at a phase delay of 90% since the ventricle ejected immediately after the pump and therefore against the systolic pressure generated by the pump ejection. It can be observed that the shape of the PV loops as well as the EW are strongly affected by the phase delay of ejection with the highest EW occurring at a phase delay of 60 to 75%.

The assistance ratio indicates a tendency towards full support conditions from 30 to 60% phase delay (Fig. 2).

Figure 3 summarizes the relevant hemodynamic parameters of three PBP experiments normalized by the maximum value for each experiment (right) to compensate for inter-experimental differences. It can be observed that the median LAP, EW, and cardiac efficiency are strongly affected by the phasing of the pulsatile pump in a sinusoidal manner – LAP was highest at the 30 to 45% delay conditions, whereas peak LVP, EW and cardiac efficiency peaked at phase delays of 60 to 75%; Oxygen consumption was highest around a 60% phase delay.

Three representative plots showing the relationships of phase delay of a PBP system with efficiency and preload are presented in Fig. 4. It was observed that preload and cardiac efficiency are not correlated (r = 0.10 ± 0.37) and are influenced in a non-linear manner: depending on the phase delay, different cardiac efficiencies were observed independent of preload.

## Discussion

In this study, a large animal isolated beating heart setup allowed the direct comparison of hemodynamics, cardiac mechanics, and energetics between LVAD support with a RBP and a PBP under well controlled hemodynamic conditions. In contrast to acute animal models, autoregulatory mechanisms such as the baroreflex and the influence of altered conditions in the venous system, right heart, and pulmonary circulation are completely decoupled. Further, the experimental setup facilitates easy application of advanced measurement systems such as a left ventricular PV loop catheter, various flow and pressure measurements, and continuous monitoring of arterial and venous oxygen content.

The ideal RBP speed setting to potentially promote myocardial recovery is a matter of debate. It is well known that increased RBP speed increases pump flow and reduces ventricular preload, as well as reduces EW and myocardial oxygen consumption^23–25^. While it is possible to fully assist the failing left ventricle with an RBP by delivering the entire cardiac output through the pump, it is clinically recognized that at least intermittent aortic valve opening is preferable for various reasons (i.e. thrombus formation, aortic valve function)^26^. Fully assisting the ventricle may also detrimentally affect myocardial reverse remodeling and recovery: A pump in full support unloads the heart but at the potential consequence of leading to atrophy of the myocardium due to excessive reduction in wall stresses. On the other hand, partial support may not reduce volume and pressure sufficiently to reverse myocardial remodeling, especially in patients with severely weak heart function. Although the ideal unloading strategy for myocardial recovery is unknown, it is commonly believed that training of the ventricle by adapting pump speed and varying the load on the ventricle may be beneficial to promote reverse remodeling and recovery^21,27^.

In this study we replicated the entire range of RBP support in isolated hearts from aggressive full to partial support conditions. We showed that with increasing RBP speed, EW is disproportionally more reduced compared to the total myocardial energy consumption, leading to a diminished cardiac efficiency in conditions of large degrees of unloading. This may be explained by the fact that during full support, the ventricular pre- and afterload decrease in tandem, leading to adverse boundary conditions for the ventricle. Based on these findings, an RBP driven at a constant speed cannot achieve a condition of a high cardiac efficiency with a simultaneously low LAP similar to that of a healthy left ventricle.

PBPs can be driven in two modes—asynchronously or synchronously to the native cardiac cycle. For asynchronously driven PBPs, the rate and the ejection volume can be prescribed to adapt the level of ventricular support. It was suggested that reloading strategies with reduction of these parameters could facilitate weaning from the device^28^; However, each heart beat supported by an asynchronously driven PBP can vary widely: dependent on the native heart rate and prescribed pump beat rate, the ventricle is loaded and/or ejects into the pump differently. Preload, afterload, EW, myocardial oxygen consumption, and therefore also cardiac efficiency is different for each beat. A synchronously driven PBP enables a nearly consistent mode of support and is adjusted by phasing of the ejection and filling of the pump—for this experiment, prescribed as the phase delay. It has previously been shown in comprehensive studies^22^ that the pump phasing has an important influence on the unloading properties of the left ventricle, however, the effect on myocardial oxygen consumption and cardiac efficiency had not yet been investigated.

In contrast to RBPs, PBPs decouple unloading and ejection by the storage of blood in the pump chamber, thereby, tailoring the extent of preload reduction and afterload adaptation independent from each other. This way, cardiac efficiency can be augmented by adjusting pump ejection and filling phases within the cardiac cycle. We showed that cardiac efficiency can be adapted by adjusting the phase delay of ejection: a ventricle supported by an adjustably phased PBP demonstrated the capacity to operate at a low preload with highest efficiencies at a phase delay of 60 to 75%. Thereby, a potential technique is granted for modifying parameters that may form ideal boundary conditions for promoting reverse cardiac remodeling and eventually cardiac recovery.

Further steps to corroborate the presented support strategy for reverse cardiac remodeling and recovery include similar experiments in chronic animal models with induced heart failure. This would be critical to prove the hypothesis that pulsatile LVAD support maintaining cardiac efficiency and a physiologic preload is beneficial for long-term reverse remodelling and recovery.

### Limitations

In this study, the contribution of the right ventricle to the myocardial oxygen consumption was kept low since the ventricle ejected only the coronary flow against a low pressure of 10 to 20 mmHg. Further, during each experiment the right ventricular output was almost constant, therefore, the relative changes in mechano-energetic parameters can be attributed to the altered loading conditions of the left ventricle. Further, healthy isolated porcine hearts were utilized which comes with several limitations: the ventricular geometry does not reflect the one of typically dilated cardiomyopathies in terms of ventricular size and wall thickness. In these experiments, although a deteriorating cardiac function over time was observed, we did not reach conditions of decompensated heart failure.

The number of experiments with the synchronized PBP was low, however, the repeatability of experiments was excellent (Fig. 4), which supports the validity of the presented findings.

## Materials and Methods

### The isolated heart setup

Porcine hearts of 8 landrace pigs (80–106 kg) were explanted similar to the protocol described previously^25^. For the explantation, a median sternotomy was performed in anesthetized pigs and the aorta was cannulated with a cardioplegic needle. Animals were exsanguinated via an aortic cannula placed in the iliac artery. As soon as 2 liters of blood were collected, the ascending aorta was clamped distal to the cardioplegic needle and 1 liter of cardioplegic solution (Custodiol, Dr. Franz Köhler Chemie GmbH, Bensheim, Germany) was applied. Simultaneously, the heart was cooled with a slurry ice solution until cardiac activity ceased.

**Figure 6:**
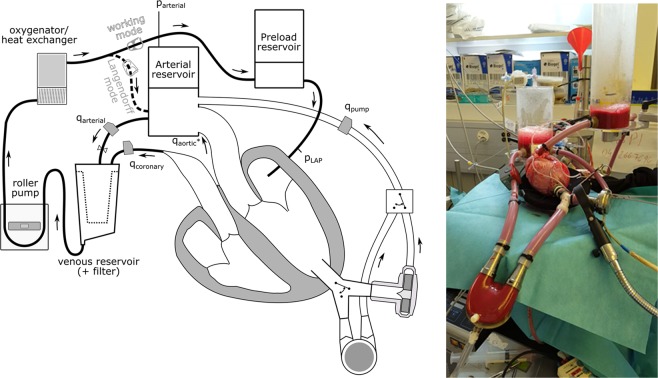
Schematic diagram and picture of the isolated heart setup in Working Mode (adapted from Granegger *et al*.^25^) with the pumps implanted.

The heart was excised and prepared for the connection to the isolated heart apparatus (Fig. 6). The right atrium was closed and a single pulmonary vein from the left atrium was left open for connection of the heart to the preload reservoir. The left ventricular apex was cored and a HVAD sewing ring (Medtronic, Minneapolis, MN, USA) was attached to the ventricle to allow pump implantation.

The isolated heart apparatus features three connections from the heart to the circuit (Fig. 6). First, the aorta was connected to an air-trapped reservoir mimicking the arterial Windkessel^29^ system. Second, the pulmonary artery was connected to an open venous reservoir so that coronary flow was ejected against a low pressure of 10 to 20 mmHg. Third, the left atrium was cannulated and connected to a pressure-controlled air-trapped reservoir which constitutes the preload adjustment system.

To resuscitate the isolated hearts, the Langendorff perfusion technique was used, where the pressure in the arterial reservoir was gradually increased to 70 mmHg. Thereby, the coronary arteries were perfused with oxygenated blood at a temperature of 37 °C. If necessary, the hearts were defibrillated to achieve a stable sinus rhythm. Once the heart stabilized, the setup was switched to the working mode, during which the heart pumps blood from the preload reservoir towards the arterial one making the left heart circulation similar to normal physiology (Fig. 6). All experiments were performed in accordance with the relevant guidelines and regulations and approved by the Ethics Committee (approval no. 219/16) of the Canton of Zurich.

To investigate the differences of RBP and PBP support, we alternately supported each ventricle with the Excor system (BerlinHeart GmbH, Berlin, Germany) or the Medtronic HVAD system. In the first 4 experiments, the Excor was driven with the BerlinHeart ExcorActive driving unit. However, this driving unit does not permit synchronized triggering to the native heart rate. Therefore, we developed our own pneumatic actuation system and an appropriate triggering and control algorithm which was used in the last 4 experiments. The developed pneumatic system consisted of an air compressor (KNF Neuberger PM21308-023.1.2, Freiburg, Germany) to provide both positive and vacuum pressure, two air tanks connected to relief valves (Niezgodka Gmbh 91-2508-4 and 1831, Hamburg, Germany), and a 5/3-way solenoid control valve (Festo MPYe-5-1/4-010-B, Esslingen am Neckar, Germany). Filling and ejection were controlled by an embedded control system developed in Simulink (Mathworks, Natick, MA, USA) and loaded onto a dSPACE MicroLabBox (dSPACE GmbH, Paderborn, Germany).

Zero-crossing detection was used to attain an accurate measurement of the sinus heartbeat duration. Using the left ventricular pressure as an input, its moving mean was subtracted from itself to generate a zero-crossing pulse signal: rising zeros occurred during ventricular systole and falling zeros during diastole. Beat duration was calculated as the duration between two rising zero crossings. The applied delays signaling for pump ejection phasing were a percentage of the beat duration and triggered from the rising zero cross point. Pump filling was then triggered at 35% of beat duration after pump ejection. Pump filling and pump ejection was passed as a state signal to a PID controller that actuated the solenoid control valves to flip the PBP from ejection and filling states. The measured pneumatic driving pressure was the process variable signal returned to the PID controller.

### Measurement protocol

Two conditions were investigated at a constant cardiac output, adjusted by the roller pump of the isolated heart setup (Fig. 6):

1. With the RBP connected, the pump speed was gradually increased to raise ventricular unloading
2. With a synchronized PBP connected, the pump was phased to eject at a delay of 0, 15, 30, 45, 60, 75, and 90%.

As soon as all parameters stabilized and at least 30 seconds after the change in speed/phase delay, the following variables were recorded for all test conditions:

1. Volume flows: aortic, pump, and coronary flow (SONOFLOW CO.55, Sonotec, Halle, Germany)
2. Pressures:

- aortic, left atrial, and pulmonary artery pressure (PAP) (APT300, Harvard Apparatus, Hollisten, MA, USA)
- left ventricular pressure (Ventri-Cath 507 PV Loop Catheter/MPVS Ultra PV Unit, ADInstruments, Sydney, Australia)
- pneumatic driving pressures of the PBP (APT300, Harvard Apparatus, Hollisten, MA, USA)

3. Left ventricular volume (LVV) (Ventri-Cath 507 PV Loop Catheter/MPVS Ultra PV Unit, ADInstruments, Sydney, Australia)
4. Arterial and venous oxygen partial pressures (P_a_O_2_, P_v_O_2_) as well as arterial and venous oxygen saturations (S_a_O_2_, S_v_O_2_) (CDI System 500, Terumo, Tokyo, Japan)
5. Hemoglobin (Hb) (CDI System 500, Terumo, Tokyo, Japan).

The gain of the left ventricular volume conductance catheter was calibrated while the heart was unsupported by matching the measured cardiac output to the measured stroke volume multiplied by the heart rate. Offset (parallel conductance) of the ventricular volume catheter was calibrated after each experiment once cardiac activity had ceased completely. By inserting a latex balloon into the ventricle and gradually increasing the volume of fluid inside, the end-diastolic pressure volume relationship was determined.

### Oxygen consumption

The oxygen content in the arterial and venous systems (C_a_O_2_, C_v_O_2_ in *ml O*_2_*/L*) were calculated from Hemoglobin (Hb in *g/L*), oxygen saturation (SO_2_ in *%)*, and partial pressure of oxygen (PO_2_ in *mmH*g).

1 $${C}_{a,v}{O}_{2}=(Hb\ast 1.39\frac{ml\,{O}_{2}}{g}\ast \frac{{S}_{a,v}{O}_{2}}{100})+(0.0031\frac{ml{O}_{2}}{L\ast mmHg}\ast {P}_{a,v}{O}_{2})$$

The myocardial oxygen consumption (MVO_2_ in *ml O*_2_*/min)* was calculated from the coronary flow (Q_cor_ in *L/min*) as:

2 $$MV{O}_{2}={Q}_{cor}({C}_{a}{O}_{2}-{C}_{v}{O}_{2}),$$

The energy consumption (P in *J/beat*) of the ventricle was calculated from the MVO_2_ and the heart rate (HR in *beats/min)*, determined based on the LVP signal, as:

3 $$P=MV{O}_{2}\ast 20.1\frac{J}{ml\,{O}_{2}}/HR,$$

Both EW and energy consumption were normalized for the heart weight and are presented in *J/beat/100* *g*.

### Delivered mechanical work

The mechanical work per beat of the ventricle was determined based on the recorded PV loops of the ventricle. The EW equals the area enclosed by PV loop for each cardiac cycle. It is the energy which is delivered to the vascular system by the left ventricle^30^.

Cardiac efficiency (in %) was defined as the ratio between the EW performed by the ventricle during each cardiac cycle and the total energy demand determined through the myocardial oxygen consumption (Eq. 3).
